# Serum Levels of Resistin, Adiponectin, and Apelin in Gastroesophageal Cancer Patients

**DOI:** 10.1155/2014/619649

**Published:** 2014-06-24

**Authors:** Dorota Diakowska, Krystyna Markocka-Mączka, Piotr Szelachowski, Krzysztof Grabowski

**Affiliations:** Department of Gastrointestinal and General Surgery, University of Medicine, Sklodowska-Curie 66, 50-369 Wroclaw, Poland

## Abstract

The aim of the study was the investigation of relationship between cachexia syndrome and serum resistin, adiponectin, and apelin in patients with gastroesophageal cancer (GEC). *Material and Methods*. Adipocytokines concentrations were measured in sera of 85 GEC patients and 60 healthy controls. They were also evaluated in tumor tissue and appropriate normal mucosa of 38 operated cancer patients. *Results*. Resistin and apelin concentrations were significantly higher in GEC patients than in the controls. The highest resistin levels were found in cachectic patients and in patients with distant metastasis. Serum adiponectin significantly decreased in GEC patients with regional and distant metastasis. Serum apelin was significantly higher in cachectic patients than in the controls. Apelin was positively correlated with hsCRP level. Resistin and apelin levels increased significantly in tumor tissues. Weak positive correlations between adipocytokines levels in serum and in tumor tissue were observed. * Conclusions*. Resistin is associated with cachexia and metastasis processes of GEC. Reduction of serum adiponectin reflects adipose tissue wasting in relation to GEC progression. Correlation of apelin with hsCRP can reflect a presumable role of apelin in systemic inflammatory response in esophageal and gastric cancer.

## 1. Introduction

Advanced malignances of esophagus, esophageal-gastric junction, and stomach are associated with weight loss, muscle atrophy, anorexia, hypercatabolism, malabsorption, and production of acute phase proteins, which lead to cancer cachexia [1, 2]. The mechanism of cancer cachexia is multifactorial and not entirely explained. Several studies show a model of development of cancer cachexia in relation to tumor-induced chronic inflammation [2–4]. In this model, the presence of low-grade tumor induces host immune reactions, which lead to the chronic inflammatory response. Both, tumor and host cells produce different mediators, for example, C-reactive protein (CRP), proinflammatory cytokines with tumor necrosis factor-*α* (TNF-*α*), interleukin-6 (IL-6), interferon-*γ* (IFN-*γ*) [3, 4], and adipocytokines [5, 6]. On the one hand, these factors have a protective role in the first phase of cancer development and on the other hand, an unlimited process of inflammation has deleterious effects. Systemic inflammatory response in advanced cancer is associated with long persisted macromolecules catabolism and in consequence with poor prognosis and shortened survival of patients [2, 3].

Adipocytokines, derived from adipose tissue, are proteins with autocrine, paracrine, and endocrine functions. They play an important role in lipid and glucose metabolism, regulation of energy balance, body homeostasis, and regulation of inflammatory processes [5–7]. Adipocytokines have been implicated in several malignances and many studies have shown their important role in development, progression, and prognosis of many types of cancer [7–10].

Adiponectin is a protective hormone, which influences anti-inflammatory, antitumor, and antiangiogenic effects [8]. Several reports have indicated the association between serum adiponectin levels and cancer cachexia presence, but these data differ in breast, lung, colon, and gastric cancer [8–11].

Resistin is secreted form adipocytes, but it is also produced by monocytes and macrophages of peripheral blood [8]. Results of previous studies suggest that resistin can exert effects, which are opposite to those exerted by adiponectin [8]. High level of serum resistin has been found in lung and colorectal cancers [8, 10].

Apelin is a peptide expressed in various tissues, including gastrointestinal tract, heart, lung, liver, and bone [12]. It has been reported in experimental and clinical studies that apelin is a mitogenic factor for the endothelial cells and stimulates tumor angiogenesis [12].

The potential role of resistin, adiponectin, and apelin in in gastroesophageal cancer (GEC) and their influence on cancer progression and cachexia syndrome are not entirely explained. The aim of the present study was the investigation of (a) possible relationship between cancer cachexia and levels of serum adiponectin, apelin, and resistin and (b) correlation of serum adipocytokines with clinical, pathological, and blood parameters of cancer patients. We analyzed also adipocytokines levels in tumor tissue and normal mucosa of patients with GEC.

## 2. Material and Methods

### 2.1. Study Population

We enrolled 145 individuals for current study: 85 patients with histologically confirmed gastroesophageal cancer (GEC) (male-to-female ratio: 61/24; mean age: 60 ± 9 years), hospitalized in the Department of Gastrointestinal and General Surgery of Wroclaw Medical University from April 2008 to December 2012, and 60 apparently healthy individuals without cancer disease serving as reference (male-to-female ratio: 47/13; mean age: 58 ± 4 years).

Patients with accompanying severe diseases, other malignancies, infections, and major operations within 6 months prior to current hospitalization were excluded from study. Samples were collected prior to any treatment. Patients were diagnosed and staged clinically on the basis of upper digestive tract endoscopy with biopsy, computer tomography, and magnetic resonance and staged pathologically after tumor resection according to the UICC TNM system [13]. There were 39 patients diagnosed with squamous cell carcinoma of esophagus, 22 with adenocarcinoma of gastroesophageal junction, and 24 with gastric adenocarcinoma. Surgical resection of tumor was carried out in 38 (44.7%) of GEC patients and palliative procedures were provided in remaining cases. Cachexia was defined as involuntary weight loss exceeding 5% of previous baseline body weight during three-month period [14]. The control group consisted of age- and sex-matched (resp., *P* = 0.161 and *P* = 0.634) healthy blood donors from the Regional Center of Blood Donation and Therapy, Wroclaw, Poland. Detailed characteristics of study population are given in Table 1.

### 2.2. Ethical Considerations

The study protocol follows ethical standards detailed in the Declaration of Helsinki, as revised in 1983, and was approved by the Medical Ethics Committee of Wroclaw Medical University, Wroclaw, Poland (number KB-784/12). Informed consent has been obtained from all study participants.

### 2.3. Analytical Methods

After overnight fasting, blood was drawn from cubical vein of untreated patients into serum-separator tubes, allowed to clot for 30 minutes, and then centrifuged (10 min, 720 ×g, RT). Collected sera were aliquoted and stored in −45°C until analysis.

Tissue sections of tumor and normal mucosa (approximately 10 cm from tumor) were taken postoperatively from 38 patients undergoing curative resection and rinsed in 0.9% NaCl. Subsequently, samples were homogenized on ice 1 : 2 (w/v) in 10 mM Tris-HCl with 1 mM EDTA, pH 7.4 buffer, and centrifuged (1850 ×g, 10 min, 4°C). The supernatants were aliquoted and stored at −45°C until analysis.

Serum and tissue levels of adiponectin and resistin were measured by immunoenzymatic assays using commercially available ELISA kits from R&D Systems (Abingdon, UK), according to manufacturer's instructions. Sensitivity of adiponectin assay was 0.246 ng/mL while intra- and interassay coefficients of variation (CV) were 2.5–4.7% and 5.8–6.9%, respectively. Sensitivity of resistin assay was 0.026 ng/mL, and intra- and interassay CVs were 3.8–5.3% and 7.8–9.2%, respectively. Apelin in sera and tissue samples was measured using ELISA assays provided by Phoenix Pharmaceuticals Inc. (Burlingame, California, USA) according to manufacturer's instructions. Assay sensitivity was 0.09 ng/mL and intra- and interassay CVs were, respectively, 5–10% and <15%.

Serum high sensitive C-reactive protein (hsCRP) was determined by immunoturbidimetric method with the Quantia-CRP UV (Tulip Diagnostics Ltd., Goa, India), in which the detection limit was 0.1 mg/mL.

Baseline blood parameters: total protein, albumin, hemoglobin, and total lymphocyte count were measured using automated procedures and obtained from Central Laboratory of the First University Hospital, Wroclaw, Poland or from Laboratory of Regional Center of Blood Donation and Therapy, Wroclaw, Poland. Body mass index (BMI) was calculated as follows: weight [kg] divided by the square of height [m]. Respective data were retrieved from medical records.

### 2.4. Statistical Analysis

Data distributions were tested with the Shapiro-Wilk normality test and homogeneity of variances was examined with Levene's test. Data were log-transformed to obtain normal distribution and, if not otherwise stated, presented as means ± SD. Differences in means were examined using one-way ANOVA with Tukey's post hoc test. Data were coexamined using two-way ANOVA (categorical data) and ANCOVA (categorical and continuous data). Frequency was analyzed using Fisher exact test or Chi-square test with Yates correction. Paired samples *t*-test was used for comparison of adipocytokines levels in tumor and matched control tissues. Correlation analysis was conducted with Pearson test. Multiple regression analysis was performed using linear regression model. Only variables with *P* value < 0.10 at univariate analysis were qualified to multivariate analysis models. The strength of associations was determined by receiver operating characteristics (ROC) analysis and expressed in terms of area under ROC curve (AUC). Sensitivity and specificity at optimal cut-off value determined as the one associated with the highest Youden index *J* were calculated as well. Two-tailed *P* value ≤ 0.05 was considered statistically significant. All analyses except for ROC were performed using Statistica 10.0 software (StatSoft Inc., Tulsa, OK, USA). ROC analysis was conducted using MedCalc Statistical Software version 13.2.2 (MedCalc Software, Ostend, Belgium; http://www.medcalc.org/; 2014).

## 3. Results

### 3.1. Demographic, Pathological, and Biochemical Characteristics of Cachectic and Noncachectic GEC Patients in Comparison to Healthy Controls

As demonstrated in Table 1, demographic and clinic-pathological characteristics of controls and GEC patients with or without cachexia showed no differences except for BMI (the lowest in cachectic cancer patients) and the disease stage distribution (higher prevalence of advanced cancers in cachectic patients).

Cancer patients had significantly lower total protein and hemoglobin concentrations and higher hsCRP levels, regardless of their cachexia status. Cachectic patients had higher albumin levels than noncachectic ones, but it was not significant (Table 2).

### 3.2. Resistin in GEC

**Figure 1:**
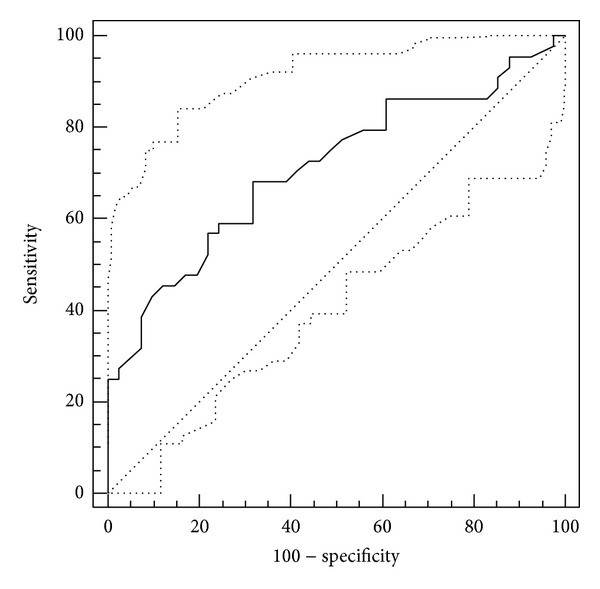
Receiver operating characteristic (ROC) analysis of serum resistin association with cachexia in GEC patients.

Serum resistin was significantly higher in cancer patients than in the controls. Resistin levels increased significantly with presence of cachexia in GEC patients (Table 2). The strength of association between serum resistin and cachexia was evaluated using ROC analysis (Figure 1). The overall accuracy of resistin as a potential indicator of cachexia was moderate (AUC = 0.71 (95% CI: 0.60–0.81), *P* < 0.001). Using 9.4 ng/mL as an optimal cut-off concentration, resistin sensitivity and specificity in discriminating cachectic from noncachectic GEC patients were 56% and 68%, respectively.

Serum resistin inversely correlated with BMI while no associations with indices of nutritional status, anemia, or inflammation could be observed (Table 3). Analysis of covariance demonstrated that cachexia status (*P* = 0.036) and not BMI (*P* = 0.286) was significantly associated with serum resistin. Among clinic-pathological variables, serum resistin levels were significantly higher in GEC patients with distant metastases (Table 4). Since there was higher prevalence of metastatic cancers in cachectic patients (*P* = 0.013), we reanalyzed the data using two-way ANOVA and found both cachexia (*P* = 0.012) and metastatic status (*P* < 0.001) to be independently associated with serum resistin.

There was a weak positive correlation between serum resistin concentrations and its levels in tumor tissue (*r* = 0.31, *P* = 0.024). Resistin content in tumor tissue was marginally higher than in the matched macroscopically normal mucosa (65.1 ± 35.5 ng/g of tissue versus 51.9 ± 32.3 ng/g of tissue, *P* = 0.048) but did not significantly correspond with cachexia status (*P* = 0.722) or any of pathological variables (*P* = 0.268 for the disease stage, *P* = 0.220 for T status, and *P* = 0.269 for N status).

### 3.3. Adiponectin in GEC

Serum adiponectin was significantly lower in cachectic GEC patients than in the controls (Table 2) and positively correlated with BMI (Table 3). No associations with histological findings were observed, but serum adiponectin levels were significantly decreased in GEC patients with metastatic disease, both regional and distant (Table 4).

Although correlation coefficient for tissue and serum adiponectin levels was tolerable (*r* = 0.56, *P* < 0.001), the differences in adiponectin levels between tumor tissue and control tissue were insignificant (4.63 ± 4.73 *μ*g/g of tissue versus 3.97 ± 2.29 *μ*g/g of tissue, *P* = 0.105). Adiponectin level in tumor tissue was not significantly associated with cachexia status (*P* = 0.943) and pathological variables (*P* = 0.067 for disease stage, *P* = 0.059 for T status, and *P* = 0.890 for N status).

### 3.4. Apelin in GEC

Serum apelin was significantly higher in GEC patients compared to healthy controls, especially in cachectic patients (Table 2). Apelin was positively correlated with hsCRP and negatively correlated with hemoglobin level (Table 3). Multiple regression analysis confirmed that hsCRP level was positively associated with serum apelin (*P* = 0.022).

Any significant relationships with clinic-pathological parameters were demonstrated, but serum apelin concentrations tended to increase in patients with esophageal squamous cell carcinoma (Table 4).

There was a weak positive correlation between serum apelin concentrations and their levels in tumor tissue (*r* = 0.30, *P* = 0.029). Apelin level in tumor tissue was somewhat higher than in the normal mucosa (22.9 ± 18.5 ng/g of tissue versus 16.9 ± 8.9 ng/g of tissue, *P* = 0.036). Tumor apelin did not significantly correspond with cachexia status (*P* = 0.262) or any of pathological variables (*P* = 0.631 for the disease stage, *P* = 0.875 for T status, and *P* = 0.980 for N status).

## 4. Discussion

In present study we demonstrated that the level of serum resistin was significantly higher in GEC patients than in the controls. This result is in agreement with previous studies, which reported that serum resistin is elevated in lung, colorectal, gastric, and esophageal cancers [8, 10, 15–19].

Resistin, as other adipocytokines, participates in regulation of systemic inflammatory response, stimulating the production of IL-6, IL-8, IL-12, and TNF-*α* in white adipose tissue [20–22]. Resistin induces growth, differentiation, and migration of endothelial cells, which is important in tumorigenesis and angiogenesis processes [16, 20, 22–24]. Our results suggest that concentrations of serum resistin can increase during cytokine-stimulated inflammatory response in GEC patients.

We observed also significantly higher levels of serum resistin in cachectic than in noncachectic patients. In addition, resistin was negatively correlated with BMI, anorexia-associated parameter. Cancer cachexia-anorexia syndrome is characterized, among other things, by decrease of calorie intake and increase of energy expenditure [1–4]. Systemic inflammatory response, with production of proinflammatory cytokines by tumor mass and immune system cells, may lead to loss of food energy acquisition, metabolic disturbances, and decrease of BMI in cancer patients [1–4, 19, 25]. Karapanagiotou et al. [15] have shown that resistin concentration increases in patients with lung cancer and weight loss. Authors suggest that resistin may contribute to the cachexia related weight loss through its participation in catabolic processes. However, Kerem et al. [16] have reported that serum resistin concentration was high in both noncachectic and cachectic gastric cancer patients. Our result of ROC analysis also indicated that importance of resistin as a marker of cachexia was not satisfactory. Despite the fact that resistin is associated with cachexia development, it cannot be used as a diagnostic marker of this process.

We have also demonstrated that serum resistin was significantly higher in GEC patients with distant metastasis. It has been shown that increased level of resistin was related to TMN stage and primary tumor progression of gastric and esophageal cancer [17, 18]. Our study is the first one, which analyzed effects of interaction between cachexia, distant metastasis, and resistin levels in GEC. We found that cachexia and metastatic status were independently associated with serum resistin. On the basis of the above-mentioned observations [15, 19] and our results, we assume that alteration of resistin level can influence systemic inflammatory response in cachexia and metastasis.

The importance of resistin in cancer cachexia appears to be different from this, which was proposed for leptin in our previous study [26]. We have demonstrated that reduction of leptin level in cancer patients may be a consequence of catabolic changes during cachexia process. However, leptin is predominantly secreted by white adipose tissue in response to various nutritional and inflammatory mediators and its low production in cachexia may be associated with adipose tissue mass degradation, while humans resistin is mainly expressed in bone marrow, trophoblastic cells of placenta, synovial tissue and fluid, epithelial cells of gastrointestinal tract, and circulating blood [20, 21]. Low level of resistin was found in white adipose tissue, in which the main source of this protein is monocytes and macrophages [21]. Steppan et al. [27] have shown that resistin-*β*, member of family of resistin-like molecules, is secreted in endothelial cells of gastrointestinal tract and is overexpressed in tumors. It suggests the possible role of this cytokine in tumorigenesis and proliferation of cancer cells [20, 27]. Tumor tissue is one of sources of proinflammatory factors. Because of that, we examined resistin level in primary tumor and normal mucosa in operated GEC patients. However, resistin level in tumor tissue was marginally higher than in the matched macroscopically normal mucosa. A weak positive correlation between serum resistin concentration and its level in tumor tissue was observed. There was no relation between tumor resistin and clinic-pathological parameters. Further studies are necessary for better clarification of the main source of resistin in GEC.

Adiponectin is a peptide hormone, which shows anti-inflammatory activity. Protective function of this protein in the development of metabolic disorders has been shown [6, 10]. In cancer, adiponectin demonstrates antiangiogenic and antitumor activities through induction of apoptosis in activated endothelial cells [10, 28–30]. Our results showed significantly lower concentrations of serum adiponectin in patients with lymph node and distant metastasis. Negative relationship between decrease of serum adiponectin level and disease progression or tumor growth in esophageal and gastric cancer has been reported [5, 6, 11, 29, 30]. These findings support the hypothesis that, in patients with advanced GEC, the expression of adiponectin may be reduced and protective actions of this peptide may be inhibited.

In our study, concentrations of serum adiponectin were significantly lower in cachectic GEC patients than in healthy subjects. Also, positive correlation between adiponectin and BMI in cancer patients was observed. This result contradicts with previous studies, which have shown that adiponectin levels increased significantly in cachectic patients with gastric and gastrointestinal cancers [16, 19] or remained unchanged in cachectic and noncachectic patients with breast, colorectal, and lung cancers [9, 31].

Adipose tissue secretes hormones, which are not connected with inflammation in cachexia [19]. Their levels reflect rather adipose tissue wasting, than active participation in cachexia-associated processes. Adiponectin represents this type of adipocytokines [8, 19]. One of the existing theories assumes that secretion of this factor may increase due to catabolic wasting process and uncontrolled increase of energy expenditure in adipose tissue during cachexia [16, 19]. However, we suggest, in our previous study, that lower production of cytokines by fat cells may be a reflection of adipose tissue devastation in relation to cachexia process [25]. Thus, catabolic reactions and uncontrolled energy consumption may contribute to adipose tissue degradation and reduction of adiponectin expression. Besides this hypothesis, it has been postulated that various cytokines, especially TNF-*α*, may inhibit secretion of adiponectin by fat cells [7, 9, 11, 32]. TNF-*α* is intensively produced by tumor cells in advanced cancer and it may suppress adiponectin expression in adipose tissue. Our results correspond to these hypotheses. To our knowledge, we demonstrated, as the first ones, that adiponectin level in tumor tissue did not differ from control mucosa. It suggests that circulating level of adiponectin reflects mainly the expression of this factor from adipose tissue in GEC patients.

Apelin is expressed in many tissues including gastrointestinal tract, heart, lung, and liver [33]. It was observed that this bioactive protein stimulates proliferation and migration of retinal endothelial cells and is required to normal vascular development [12, 34]. Apelin has been shown as a potentially important proangiogenic factor in cancers [12, 33–35].

We demonstrated that serum apelin level was significantly higher in GEC patients than in healthy controls, especially in cachectic patients. Our study did not show significant associations between apelin levels and clinic-pathological parameters of cancer patients. We observed tendency to the highest levels of apelin concentration in patients with esophageal squamous cell carcinoma in comparison to patients with gastric adenocarcinoma. Esophageal squamous cell cancer is very aggressive with rapid primary tumor growth and early metastasis to the regional lymph node [26]. Increased level of apelin in this type of cancer may correlate with tumor angiogenesis.

Additionally, we showed a significantly higher hsCRP level and significantly lower concentrations of total protein, albumin, and hemoglobin in cancer patients. Among cancer patients, we as the first ones demonstrated positive correlation between apelin and hsCRP levels and negative correlation between apelin and hemoglobin levels. Our previous study showed that serum hsCRP levels increased in the presence of regional lymph node metastasis in GEC patients [36]. All of the mentioned results suggest that apelin production is probably related to systemic inflammatory response in GEC patients.

In conclusion, resistin is associated with cachexia and metastasis processes of GEC. Reduction of serum adiponectin reflects adipose tissue wasting in relation to GEC progression. Correlation of apelin with hsCRP can reflect a presumable role of apelin in systemic inflammatory response in esophageal and gastric cancer.

## Figures and Tables

**Table 1:** Characteristics of study population.

2008–2012 years	Healthy controls (*n* = 60)	Gastroesophageal cancer		*P*-value
		Noncachexia (*n* = 41)	Cachexia (*n* = 44)	
Age (years)	58.1 ± 4.2	61.8 ± 11.9	60.2 ± 9.0	0.780^a^
Gender (male/female)	47/13	27/14	35/9	0.156^b^
BMI (kg/m^2^)	26.7 ± 2.7	24.9 ± 1.7	20.3 ± 2.2	<0.001^a^
Localization				
Esophagus	—	18 (43.9)	21 (47.7)	0.939^b^
Cardia	—	11 (26.8)	11 (25.0)	
Gaster	—	12 (29.3)	12 (27.3)	
Operability (operable/not operable)	—	22/19	15/29	0.069^b^
Histological type				
Squamous cell carcinoma	—	18 (43.9)	21 (47.7)	0.724^b^
Adenocarcinoma	—	23 (56.1)	23 (52.3)	
TNM stage				
II	—	10 (24.4)	0 (0.0)	0.002^b^
III	—	12 (29.3)	15 (34.1)	
IV	—	19 (46.3)	29 (65.9)	

Data presented as mean ± SD or sizes (percent, %).

^a^One-way ANOVA test, ^b^chi-square, or Fisher exact tests.

**Table 2:** Biochemical and blood parameters in study groups.

Parameter	Healthy controls (*n* = 60)	GEC patients		*P*-value
		Noncachectic (*n* = 41)	Cachectic (*n* = 44)	
Hemoglobin (g/L)	15.9 ± 1.3^a,b^	12.3 ± 1.8^c^	11.8 ± 2.0^c^	<0.001∗
Lymphocytes (10^9^/L)	2.2 ± 1.3	2.9 ± 1.7	2.8 ± 1.2	0.365
Total protein (g/L)	69.9 ± 5.9^a,b^	58.2 ± 7.1^c^	58.2 ± 10.7^c^	<0.001∗
Albumin (g/L)	39.2 ± 4.0^a^	35.0 ± 7.2^c^	37.5 ± 4.8	0.039∗
hsCRP (mg/L)	1.05 ± 0.65^a,b^	108.1 ± 53.3^c^	110.3 ± 82.9^c^	<0.001∗
Resistin (ng/mL)	7.5 ± 2.7^b^	8.99 ± 3.21^b^	11.74 ± 2.98^a,c^	<0.001∗
Adiponectin (*μ*g/mL)	9.81 ± 4.1^b^	8.86 ± 3.79	8.02 ± 4.10^c^	0.248
Apelin (pg/mL)	635 ± 365^b^	820 ± 211	855 ± 195^c^	0.014∗

Data presented as means ± SD and analyzed using ANOVA with post hoc Tukey test.

*Statistically significant; ^a^significantly different from noncachectic cancer patients; ^b^significantly different from cachectic cancer patients; ^c^significantly different from controls.

**Table 3:** Relationship between serum adipocytokines and patient's gender, age, BMI, and blood parameters.

	Resistin	Adiponectin	Apelin
Gender			
*P*	0.305	0.207	0.421
Age			
*r*	0.013	0.024	0.129
*P*	0.937	0.885	0.239
BMI			
*r*	−0.364	0.350	−0.173
*P*	<0.001∗	<0.001∗	0.113
Total protein			
*r*	−0.079	0.183	−0.080
*P*	0.472	0.093	0.466
Albumin			
*r*	−0.062	0.092	−0.084
*P*	0.572	0.402	0.444
hsCRP			
*r*	0.137	0.032	0.280
*P*	0.211	0.771	0.009∗
Hemoglobin			
*r*	−0.081	0.077	−0.230
*P*	0.461	0.484	0.034∗
Lymphocytes			
*r*	0.081	0.128	0.205
*P*	0.461	0.243	0.059

*r*, Pearson correlation coefficient; *statistically significant.

**Table 4:** Relationship between clinic-pathological parameters and serum levels of resistin, adiponectin, and apelin in GEC patients.

	Resistin (ng/mL)		Adiponectin (*μ*g/mL)		Apelin (pg/mL)	
	mean ± SD	*P*	mean ± SD	*P*	mean ± SD	*P*
Histological type		0.495		0.277		0.065
scc (*n* = 39)	10.9 ± 3.3		9.02 ± 4.33		886 ± 127	
adca (*n* = 46)	10.4 ± 3.4		8.08 ± 3.59		836 ± 118	
TNM stage		0.223		0.260		0.381
II (*n* = 10)	9.2 ± 3.3		9.3 ± 3.8		889 ± 117	
III (*n* = 27)	10.7 ± 2.7		7.9 ± 4.8		818 ± 176	
IV (*n* = 48)	11.2 ± 3.6		7.2 ± 2.5		862 ± 199	
Tumor stage (T)		0.330		0.484		0.231
T2 (*n* = 11)	9.1 ± 3.3		8.8 ± 3.7		801 ± 135	
T3 (*n* = 22)	9.9 ± 3.6		8.3 ± 6.4		828 ± 160	
T4 (*n* = 52)	11.1 ± 3.2		7.4 ± 1.2		891 ± 154	
Lymph node metastasis		0.142		0.012∗		0.104
N0 (*n* = 26)	10.2 ± 2.9		9.5 ± 3.7		821 ± 146	
N1 (*n* = 59)	11.3 ± 3.7		7.4 ± 3.8		865 ± 101	
Distant metastasis		<0.001∗		0.037∗		0.106
M0 (*n* = 38)	9.6 ± 3.1		9.5 ± 4.1		836 ± 152	
M1 (*n* = 47)	12.2 ± 3.2		7.7 ± 3.6		881 ± 101	

Data analyzed using one-way ANOVA or *t*-test for independent samples. scc: squamous cell carcinoma; adca: adenocarcinoma; *statistically significant.
